# Hyaluronidase treatment is associated with increased cytokine levels and reduced peritoneal neutrophil accumulation during LPS-induced inflammation

**DOI:** 10.3389/fphar.2026.1869630

**Published:** 2026-08-21

**Authors:** Karla de Castro Figueiredo Bordon, Karina Furlani Zoccal, Sayuri Noemi Otuka, Mouzarllem Barros Reis, Cryslane Almeida de Lima, Ualter Guilherme Cipriano, Vania Luiza Deperon Bonato, Eliane Candiani Arantes

**Affiliations:** 1 Department of BioMolecular Sciences, School of Pharmaceutical Sciences of Ribeirão Preto, University of São Paulo (USP), Ribeirão Preto, Brazil; 2 Barão de Mauá University Center (CUBM), Ribeirão Preto, Brazil; 3 Basic and Applied Immunology Program, Department of Biochemistry and Immunology, Ribeirão Preto Medical School, University of São Paulo (USP), Ribeirão Preto, Brazil

**Keywords:** drug discovery, extracellular matrix, glycocalyx, hyaluronic acid, innate immunity, leukocyte trafficking, translational pharmacology, venom-derived therapeutics

## Abstract

**Background:**

Hyaluronidases are classically described as spreading factors that facilitate venom dissemination through extracellular matrix degradation, but their immunomodulatory roles remain poorly defined. In particular, comparative data between native scorpion venom hyaluronidase and commercial bovine hyaluronidase remain limited. This study investigated whether hyaluronidase treatment was associated with condition-dependent changes in inflammatory parameters.

**Methods:**

BALB/c mice were subjected to lipopolysaccharide (LPS)-induced peritoneal inflammation and treated with a native *Tityus serrulatus* hyaluronidase (TsHyal-1) or bovine PH20. Leukocyte recruitment was evaluated by total and differential cell counts. Inflammatory mediators were quantified in peritoneal lavage fluid. Histopathological analyses of liver, lung, and kidney were performed using hematoxylin and eosin staining. Biochemical characterization of both enzymes was performed in parallel with the evaluation of inflammatory and histological parameters to compare their inflammatory profiles under distinct inflammatory conditions.

**Results:**

Under non-LPS conditions, both TsHyal-1 and PH20 increased neutrophil recruitment and IL-6/IL-1β levels, accompanied by mild to moderate tissue alterations. In contrast, during LPS-induced peritoneal inflammation, both enzymes induced a non-canonical inflammatory profile in which IL-6/IL-1β levels were increased while neutrophil recruitment was reduced. TsHyal-1 exhibited a more pronounced immunomodulatory profile than bovine PH20. These differences were observed despite equivalent catalytic activity and nearly molar dosing and may reflect intrinsic biochemical or structural characteristics of the enzymes.

**Conclusion:**

Treatment with TsHyal-1 and bovine PH20 was associated with context-dependent changes in inflammatory parameters in this experimental model. These findings expand current knowledge of hyaluronidase biology and provide a basis for future studies investigating their role in inflammatory regulation under defined inflammatory contexts.

## 1 Introduction

Acute inflammatory conditions, including sepsis-associated inflammation, remain leading causes of organ failure and mortality worldwide (He et al., 2021; Srdić et al., 2024). Despite advances in critical care, effective strategies to control dysregulated inflammation in sepsis remain limited, underscoring the need for approaches targeting host responses and extracellular environment interactions (Jain et al., 2024; Papatheodosiou et al., 2026). The extracellular matrix (ECM) is increasingly recognized as a key regulator of inflammatory and immunological responses. Hyaluronan (HA), a major ECM component, illustrates this functional duality: high-molecular-weight HA (HMW-HA) promotes tissue integrity and immunological homeostasis, while low-molecular-weight fragments (LMW-HA) generated during inflammation act as damage-associated molecular patterns (DAMPs) that engage pattern recognition receptors and have been reported to promote leukocyte recruitment (Sokolowska et al., 2014). Clinical observations from *Bothrops* envenomation further suggest that extracellular proteolytic balance may influence inflammatory outcomes (Ferreira Neves et al., 2026).

Beyond the canonical HMW-HA/LMW-HA paradigm, the ECM contributes to immune regulation through dynamic changes in the extracellular environment that influence leukocyte trafficking, cytokine availability, and receptor signaling (Lu et al., 2025). In this context, ECM remodeling extends beyond HA fragmentation and includes broader changes in the extracellular environment that may influence inflammatory responses (Silva de França and Tambourgi, 2023).

Venom-derived enzymes have emerged as potent modulators of host inflammatory responses, in addition to their classical roles as spreading factors (Rao et al., 2024; Salazar et al., 2024; Silva de França and Tambourgi, 2023). Hyaluronidases have been reported to influence leukocyte migration, vascular permeability, and cytokine signaling (Balduit et al., 2023). One proposed mechanism involves the cleavage of high-molecular-weight HA into bioactive low-molecular-weight fragments, which have been reported to activate immune cells via TLR2 and TLR4-dependent pathways (Silva de França and Tambourgi, 2023). However, beyond HA fragmentation, hyaluronidases have been reported to modify the extracellular environment through multiple complementary mechanisms, including disruption of the endothelial glycocalyx (van der Heijden et al., 2025), facilitation of tissue diffusion (de Oliveira-Mendes et al., 2019; Silva de França and Tambourgi, 2023), and changes in the extracellular environment that may influence inflammatory signaling (Lu et al., 2025). These integrated effects on the extracellular microenvironment may collectively shape inflammatory responses. In this context, venom-derived enzymes represent an underexplored source of bioactive molecules with potential applications in immunomodulation and drug development.

The hyaluronidase from *Tityus serrulatus* venom (TsHyal-1) has been biochemically characterized and shown to contribute to venom spreading and systemic toxicity *in vivo* (Horta et al., 2014; de Oliveira-Mendes et al., 2019; Pessini et al., 2001). However, the direct impact of scorpion venom hyaluronidase on inflammatory mediators and HA biology remains incompletely characterized, particularly under systemic inflammatory conditions. Whether TsHyal-1 influences HA biology and the extracellular environment during LPS-induced peritoneal inflammation remains unknown.

In this study, we investigated the effects of *T. serrulatus* hyaluronidase on peritoneal inflammation, with emphasis on inflammatory responses associated with the extracellular environment in an LPS-induced peritoneal inflammation model. Using a commercially available bovine testicular hyaluronidase (PH20) as a reference, we compared PBS control, LPS-challenged, and enzyme-treated groups. Given that hyaluronidases have been reported to influence the extracellular environment via HA degradation and associated changes in ECM organization (de Oliveira-Mendes et al., 2019; Silva de França and Tambourgi, 2023), we hypothesized that scorpion venom hyaluronidase modulates inflammatory responses in an environment-dependent manner, an effect that may not necessarily depend on detectable HA fragmentation.

## 2 Materials and methods

### 2.1 Hyaluronidase characterization

Collection, husbandry, and venom extraction of *T. serrulatus* were conducted in accordance with institutional guidelines, Brazilian legislation, and international standards for arthropod care. Scorpion specimens were collected under IBAMA registration No. 1506748. All procedures complied with the Nagoya Protocol and Brazilian regulations governing access to genetic heritage (SISGEN registration No. A4A9FDD). Native hyaluronidase TsHyal-1 from *T. serrulatus* scorpion venom was purified following the protocol originally described by Pessini et al. (2001) based on sequential ion-exchange chromatography on CM-cellulose-52. Enzymatic activity was confirmed before the enzyme was used in all experiments. A commercially available bovine testicular hyaluronidase (PH20 - H3884; Sigma-Aldrich, St. Louis, MO, United States) was used as a reference enzyme for comparative analyses.

Specific activity of both enzymes was determined using a turbidimetric assay based on hyaluronic acid degradation (Pukrittayakamee et al., 1988). Enzymatic activity was expressed as Turbidimetric Reduction Units (TRU), defined as the amount of enzyme required to hydrolyze 50% of the hyaluronan substrate under the assay conditions. According to Pessini et al. (2001), 0.153 TRU corresponds to 1 National Formulary Unit (NFU).

For the *in vivo* experiments, each animal received 11 TRU of hyaluronidase. This dose was selected based on previous studies demonstrating *in vivo* biological activity of hyaluronidase *in vivo* over the range of 16–32 TRU, including enhancement of venom diffusion and modulation of inflammatory responses (Bordon et al., 2012; Fronza et al., 2014). Using the conversion proposed by Pessini et al. (2001), the administered dose corresponds to approximately 72 NFU per animal.

Because hyaluronidases are enzymes, dose normalization was based primarily on catalytic activity (TRU), which was considered the most appropriate parameter for comparing enzymatic preparations. To complement activity-based normalization, the administered protein mass and molar amounts were calculated from absorbance at 280 nm with protein-specific extinction coefficients predicted by ProtParam (ExPASy). Following reconstitution, the final enzyme preparations exhibited nearly identical molar concentrations (0.89 μM for TsHyal-1 and 0.91 μM for bovine PH20) (Supplementary Table S1), indicating that both enzymes were tested under equivalent catalytic activity and comparable molar concentrations. This approach minimized differences attributable to protein quantity and allowed direct comparison of the biological effects of the two hyaluronidases. Each experimental group included five animals per independent experiment, resulting in a total of ten animals per experimental condition across both experiments. A single batch of each enzyme preparation was generated in sufficient quantity to ensure consistent dosing across the study. The calculated enzyme amounts were dried under vacuum using a SpeedVac concentrator (Thermo Scientific, Waltham, MA, United States) and stored at −20 °C until use. On the day of the experiments, samples were reconstituted in phosphate-buffered solution (PBS, pH 7.4). Prior to administration, all solutions were prepared under laminar flow and filtered through 0.22 μm polyvinylidene fluoride (PVDF) membranes. Although 0.22 μm filtration does not remove endotoxin, it was performed to minimize microbial and particulate contamination. Reconstituted enzymes were then injected intraperitoneally according to the experimental protocol.

### 2.2 *In silico* physicochemical analysis of hyaluronidases

The primary amino acid sequences of hyaluronidase TsHyal-1 from *T. serrulatus* venom and bovine testicular hyaluronidase (PH20) were retrieved from the UniProt database (accession numbers P85841 and Q7YS45, respectively). Physicochemical properties, including molecular weight, theoretical isoelectric point (pI), instability index, aliphatic index, and grand average of hydropathicity (GRAVY), were calculated by the ProtParam tool available at the ExPASy server (https://web.expasy.org/protparam/). These computed physicochemical descriptors were compared between TsHyal-1 and PH20 to identify sequence-derived differences potentially related to their physicochemical properties. Sequence alignment between TsHyal-1 and bovine PH20 was generated using the MultAlin Interface Page (http://multalin.toulouse.inra.fr/multalin/) (Corpet, 1988) and visualized with the ESPript server (https://espript.ibcp.fr/ESPript/cgi-bin/ESPript.cgi) (Gouet, 2003), while sequence identity and similarity were calculated by the LALIGN algorithm (https://www.ebi.ac.uk/jdispatcher/psa/lalign) (Madeira et al., 2024). Prediction of N-glycosylation sites was carried out using the NetNGlyc 1.0 server (Technical University of Denmark, https://services.healthtech.dtu.dk/services/NetNGlyc-1.0/), which predicts potential N-linked glycosylation sites based on the consensus Asn-X-Ser/Thr (N-X-S/T) motif using artificial neural networks (Gupta and Brunak, 2002). Only canonical N-X-S/T sequons were considered for analysis. These analyses were performed as complementary computational approaches to support interpretation of the experimental findings.

### 2.3 Animals

Male and female BALB/c mice (6–8 weeks old; approximately 20 g) were obtained from the animal facility of Barão de Mauá University Center (Ribeirão Preto, São Paulo, Brazil) from an in-house breeding colony originally derived from The Jackson Laboratory and maintained under specific pathogen-free (SPF) conditions. Two independent experiments were conducted by different investigators, each including six experimental groups with five animals per group. A total of 60 mice (30 males and 30 females) were used across both experiments, with balanced sex distribution within each experimental group (2–3 males and 2–3 females per group in each experiment). Unless otherwise stated, all analyses used samples from all animals (n = 10 per experimental group; total n = 60). Cytokine quantification was carried out in a randomly selected subset of animals (n = 5 per experimental group; total n = 30), as described below. Animals were housed under standard laboratory conditions with controlled temperature and humidity, a 12 h light/dark cycle, and free access to water and food. All experimental procedures were approved by the Ethics Committee on Research and Animal Experimentation of Barão de Mauá University Center (Protocol #562/25) and were conducted in accordance with applicable guidelines for the care and use of laboratory animals.

### 2.4 Evaluation of leukocyte influx into the peritoneal cavity

Six experimental groups of mice were included in each independent experiment (n = 5 per group per experiment; final n = 10 animals per experimental group across both independent experiments): 1) PBS (control): PBS + PBS; 2) TsHyal-1 alone: TsHyal-1 + PBS; 3) PH20 alone: PH20 + PBS; 4) LPS alone: LPS + PBS; 5) LPS + TsHyal-1: LPS + TsHyal-1; 6) LPS + PH20: LPS + PH20. For all groups, the first intraperitoneal (i.p.) administration (300 μL) consisted of sterile phosphate-buffered saline (PBS), *Escherichia coli* LPS (100 μg/kg; Sigma-Aldrich, St. Louis, MO, United States), PH20 or TsHyal-1 (11 TRU/mouse), according to group allocation. Fifteen minutes later, a second i.p. injection (300 μL) was given: PBS was injected to the control, TsHyal-1 alone, PH20 alone, and LPS alone groups, whereas LPS-challenged animals received TsHyal-1 or PH20 (11 TRU/mouse).

Sixty minutes after the second dose, animals were anesthetized by i.p. treatment of ketamine hydrochloride (80 mg/kg) and xylazine (10 mg/kg). Animals were subsequently euthanized by i.p. overdose of ketamine hydrochloride (240 mg/kg) and xylazine (30 mg/kg). Peritoneal lavage was then obtained using a standardized lavage protocol by injecting 3 mL of sterile PBS into the peritoneal cavity, as previously described (Martins et al., 2024; Zoccal et al., 2025). The abdomen was gently massaged for 1 min, and lavage fluid was collected using a syringe with a needle inserted into the inguinal region. Total leukocyte counts were determined in Türk’s solution using a Neubauer chamber. Differential leukocyte counts were performed on cytospin preparations stained with a Romanowsky-based kit (Panótico® Laborclin, Paraná, Brazil). Cell counts were carried out for all animals included in both independent experiments (n = 10 per experimental group; total n = 60). For each animal, a total of 100 leukocytes were counted per cytospin slide under light microscopy and classified as neutrophils or mononuclear cells following standard morphological criteria. After centrifugation (400 *× g*, 4 °C, 10 min), cell-free peritoneal fluids were stored at −80 °C for subsequent cytokine quantification. Leukocyte counts were obtained directly from aliquots of the recovered lavage fluid and were not normalized to the recovered volume.

The LPS dose (100 μg/kg) was selected as a non-lethal inflammatory stimulus capable of inducing a measurable inflammatory response. Previous studies using murine models of LPS-induced peritonitis have demonstrated that a wide range of doses can trigger leukocyte recruitment and cytokine production, varying from nanogram amounts per animal to milligram per kilogram doses depending on the experimental design (Miyazaki et al., 2004).

### 2.5 Inflammatory mediators

Levels of IL-6 (assay range: 1,000 pg/mL to 15.6 pg/mL) and IL-1β (assay range: 1,000 pg/mL to 15.6 pg/mL) were quantified in cell-free peritoneal fluid by enzyme-linked immunosorbent assay (ELISA) in duplicate with commercially available kits (R&D Systems, Minneapolis, United States; catalog #M6000B for IL-6 and #MLB00C for IL-1β), using specific purified and biotinylated antibodies and recombinant cytokine standards according to the manufacturer’s instructions. Absorbance was measured at 450 nm and corrected at 570 nm using a VersaMax microplate reader (Molecular Devices). Cytokine quantification was carried out using peritoneal lavage samples from a randomly selected subset of animals corresponding to one-half of the total experimental population (n = 5 per experimental group; total n = 30). The subset was selected according to the predefined experimental design while maintaining balanced representation of the two independent experiments (2–3 animals from each experiment per group). The specimens were analyzed individually and were not pooled.

### 2.6 Histological processing

Lungs, liver, and kidneys were collected from euthanized animals and fixed in 10% formaldehyde. Tissues were routinely processed, embedded in paraffin blocks, sectioned (5 μm), and stained with hematoxylin and eosin (H&E). Histological analyses were conducted using Leica Qwin software (Leica Imaging Systems Ltd., Cambridge, England) coupled to a Leica DMR light microscope (Leica Microsystems GmbH, Wetzlar, Germany) equipped with a video camera (Leica Microsystems Ltd., Heerbrugg, Switzerland). All images were acquired using a 40× objective under the same imaging parameters to ensure comparability across groups.

Histological sections were evaluated qualitatively by two independent observers blinded to experimental group allocation. Histological evaluation was performed for all animals included in the study (n = 10 per experimental group; total n = 60). The analysis focused on identifying and describing tissue alterations, including inflammatory changes and morphological abnormalities. Because the primary objective of the histological assessment was to characterize tissue alterations rather than quantitative severity evaluation, a semiquantitative histological scoring system was not applied. Morphometric analyses were conducted for selected histological parameters; however, no significant differences were detected among groups, and these measurements were not used for biological interpretation. Representative images were selected based on the predominant histopathological features identified in each experimental group.

### 2.7 Electrophoretic profiling of peritoneal lavage proteins

For electrophoretic profiling, protein content was estimated by UV-Vis spectrophotometry (NanoDrop 2000; Thermo Scientific, Wilmington, DE, United States) to standardize sample loading. Electrophoretic analyses were conducted using peritoneal lavage samples from the same randomly selected subset of animals used for cytokine quantification (n = 5 per experimental group; total n = 30). Equivalent protein amounts (5 µg per sample) were loaded per lane in a final volume of 9 μL, consisting of 6 µL of protein sample and 3 µL of 3× concentrated sample buffer. After electrophoresis and staining, gels were imaged with a Gel Doc™ EZ Imager (Bio-Rad, Hercules, CA, United States).

#### 2.7.1 Polyacrylamide gel electrophoresis (PAGE)

Native PAGE was used to assess charge-dependent changes in the protein profile under non-denaturing conditions, allowing the detection of shifts in electrophoretic mobility associated with inflammation-induced alterations in the extracellular proteome. Proteins were separated under non-denaturing conditions, following the method of Heukeshoven and Dernick (1992), with minor adaptations. Polyacrylamide gels (12% in 1 M Tris-HCl, pH 8.9) were run in Tris-glycine buffer (0.4 M Tris, 0.1 M glycine, pH 8.6). Samples were diluted in native sample buffer containing 50 mM Tris-HCl, pH 8.9, 8% glycerol, and 0.1% Tween 20. No reducing agents or denaturants were added. Bromophenol blue (0.004%) was used as tracking dye. Protein standards (albumin, casein, and trypsin inhibitor) were loaded at 0.5 µg per lane. Peritoneal lavage samples were applied at 5 µg per lane (maximum volume: 4 µL). Electrophoresis was conducted at 130 V (107–72 mA) for 1 h 15 min at room temperature, until the tracking dye reached the lower region of the gel. After separation, gels were fixed in a solution containing 50% methanol, 10% acetic acid, and 100 mM ammonium acetate for 15 min, then stained with 0.02% Coomassie G-250 and documented using a gel imaging system.

#### 2.7.2 SDS-PAGE and tricine SDS-PAGE

Sodium dodecyl sulfate-polyacrylamide gel electrophoresis (SDS-PAGE) was carried out using 10% (w/v) resolving gels and 5% (w/v) stacking gels. Samples (10 µL) were prepared in final sample buffer containing 0.0625 M Tris-HCl (pH 6.8), 2% (w/v) SDS, 10% (v/v) glycerol, 5% (v/v) 2-mercaptoethanol, and 0.001% (w/v) bromophenol blue (Laemmli, 1970). Electrophoresis was carried out under denaturing conditions in a Tris-glycine system at 60 V for 30 min, and then 100 V for 1 h 45 min at room temperature. Protein bands were visualized by staining with 0.02% Coomassie Blue G-250.

For enhanced resolution of low-molecular-weight proteins, Tris-Tricine SDS-PAGE was run under denaturing conditions. Equal amounts of total protein (5 µg per lane), estimated by NanoDrop spectrophotometry (absorbance at 280 nm, using an extinction coefficient of 1.0), were adjusted to a final volume of 20 µL and mixed with 10 µL of 3× concentrated sample buffer containing SDS and a reducing agent. Samples were heated at 95 °C for 5 min, and 10 µL were loaded per lane. Gels consisted of a 10% T, 6% C resolving gel overlaid with a 5% stacking gel (Jiang et al., 2016; Schägger, 2006). Electrophoresis was performed at 60 V until entry into the resolving gel, after which the voltage was increased to 100 V until completion. Molecular mass standards included Precision Plus Protein™ Dual Color Standards (10–250 kDa; Bio-Rad, Hercules, CA, USA #161-0374) and an ultralow-molecular-weight marker (1.06–26.6 kDa; Sigma-Aldrich, St. Louis, MO, United States M3546), allowing accurate mass estimation across a broad range. Protein bands were detected by silver staining (Schägger, 2006), providing high sensitivity for low-abundance species.

### 2.8 Hyaluronan analysis in peritoneal lavage fluid

Hyaluronan (HA) was analyzed using peritoneal lavage samples from the same randomly selected subset of animals used for cytokine quantification and electrophoretic analyses (n = 5 per experimental group; total n = 30). HA was purified, quantified, and analyzed after selective enrichment of glycosaminoglycans (GAGs). The workflow combined proteolytic digestion and differential precipitation to promote dissociation of protein-HA complexes and recovery of HA in the soluble fraction. Detailed fraction classification and processing workflow are provided in Supplementary Material.

#### 2.8.1 Protein precipitation and hyaluronan enrichment

Peritoneal lavage samples (500 μL; ∼350 µg total protein) from the selected subset of animals (n = 5 per experimental group) were initially processed as individual biological replicates. Samples were subjected to papain digestion (1:20 enzyme-to-protein ratio) in PBS containing EDTA (2 mM) and cysteine (5 mM), with sequential incubation at 37 °C for 15 min and 55 °C for 15 h. Proteins were precipitated with trichloroacetic acid (10%, 4 °C–8 °C, 30 min) and removed by centrifugation (10,000 × g, 4 °C, 15 min). GAGs were subsequently precipitated from the supernatant with ethanol (three volumes, −20 °C, 20 h, 0.3 M NaCl), and centrifuged under the same conditions. Because HA recovery from individual samples was consistently low, equal aliquots from animals within each experimental group were pooled and reprocessed under the same precipitation conditions, including extended incubation (−80 °C, 64 h), to improve HA recovery and generate secondary fractions. All pellets were resuspended in ultrapure water, concentrated, and desalted using 3 kDa MWCO centrifugal filters. Retained fractions (>3 kDa) were dried and resuspended for downstream analyses.

#### 2.8.2 Spectrophotometric quantification of hyaluronan

HA was quantified using the cationic dye Stains-all (E9379, Sigma-Aldrich, St. Louis, MO, United States), based on spectral shift upon binding to non-sulfated GAGs as described by Fagnola et al. (2009). Reactions (200 µL final volume) contained sample, ultrapure water, and dye (0.03 mg/mL), and were incubated for 30 min at room temperature, protected from light. Absorbance was measured at 640 nm. Calibration curves were generated using HA standards (0.1–24 μg/mL), with excellent linearity (R^2^ ≥ 0.99). HA concentrations were determined by interpolation within the linear range and expressed as total content per experimental group.

#### 2.8.3 Electrophoretic analysis of hyaluronan

HA-enriched fractions were analyzed by PAGE under non-denaturing conditions (15% acrylamide) in Tris-borate-EDTA buffer, according to Ikegami-Kawai and Takahashi (2002). Electrophoresis was performed using a stepped voltage protocol (70 V followed by 130 V). HA standards and enzymatically fragmented HA (bovine hyaluronidase-treated) were included as references. GAGs were detected by Stains-all staining under light-protected conditions, and migration profiles were compared across groups. Additional control gels containing purified GAG standards, including hyaluronan (H1876 – hyaluronic acid sodium salt from human umbilical cord), chondroitin sulfate (C4S; CS-A; C6737 – chondroitin sulfate sodium salt from bovine cartilage; 57% C4S and 43% C6S), dermatan sulfate (DS; CS-B; C3788 – chondroitin sulfate B sodium salt from porcine intestinal mucosa), and chondroitin-6-sulfate (C6S; CS-C; C4384 – chondroitin 6-sulfate sodium salt from shark cartilage) (Sigma-Aldrich, St. Louis, MO, United States), were used to validate migration behavior and staining specificity (Supplementary Figure S3).

### 2.9 Statistical analyses

Data are presented as the mean ± standard deviation (SD). Samples presenting predefined technical problems during processing or analysis were excluded before statistical analysis. Prior to the parametric analyses, normality was assessed using the Shapiro-Wilk test, and homogeneity of variances was evaluated using the Brown-Forsythe test. Statistical analyses were performed using one-way analysis of variance (ANOVA) followed by Dunnett’s multiple-comparisons test, as specified in the corresponding figure legends. Each biological endpoint (leukocyte counts, cytokine levels, histological parameters, and hyaluronan determination) was analyzed independently according to the predefined experimental design. Multiple-comparison adjustment was performed within each endpoint using Dunnett’s multiple-comparisons test. Animals were randomly allocated to the experimental groups, and data analysis was conducted by investigators blinded to group allocation. Differences were considered statistically significant at *p* < 0.05. The sample size for each experimental endpoint is indicated in the corresponding figure legends. Leukocyte counts and histological analyses included data from two independent experiments (total n = 10 animals per group). Cytokine quantification by ELISA, electrophoretic analyses, and hyaluronan (HA) determination were carried out using n = 5 biological samples per group obtained from the two independent experiments, according to the predefined experimental design.

## 3 Results

### 3.1 *In silico* physicochemical comparison of TsHyal-1 and PH20

*In silico* analysis revealed distinct physicochemical profiles between scorpion TsHyal-1 and bovine PH20 (Supplementary Table S2). TsHyal-1 displayed a lower molecular weight (44.5 kDa) than PH20 (49.7 kDa) and a slightly higher theoretical pI (8.75 vs. 8.34). The instability index was lower for TsHyal-1 (35.02) than for PH20 (37.56), suggesting slightly greater predicted structural stability. Both enzymes exhibited high aliphatic indices and contained twelve cysteine residues, corresponding to six predicted disulfide bonds. TsHyal-1 showed a more negative grand average of hydropathicity (GRAVY) value (−0.407 vs. −0.288), consistent with slightly higher predicted hydrophilicity. Overall, the two enzymes displayed only modest differences in their predicted physicochemical characteristics.

### 3.2 Pairwise sequence comparison of TsHyal-1 and PH20

Pairwise sequence alignment revealed 34.3% amino acid identity and 67.7% similarity over a 350-residue overlap between TsHyal-1 and bovine PH20 (Supplementary Figure S4). Catalytic residues were conserved in both sequences, whereas sequence divergence was predominantly observed in peripheral regions, including differences in the distribution of cysteine residues, predicted N-linked glycosylation sites, and surface-exposed loops. Insertions and deletions were largely confined to non-catalytic regions. Both proteins contained twelve cysteine residues, although their distribution differed between the two sequences. Prediction of N-linked glycosylation sites identified four sites in TsHyal-1 and six in bovine PH20 (Supplementary Table S2; Supplementary Figure S4).

### 3.3 Hyaluronidase differentially modulates leukocyte recruitment, reducing neutrophil accumulation under LPS-stimulated conditions

**FIGURE 1:**
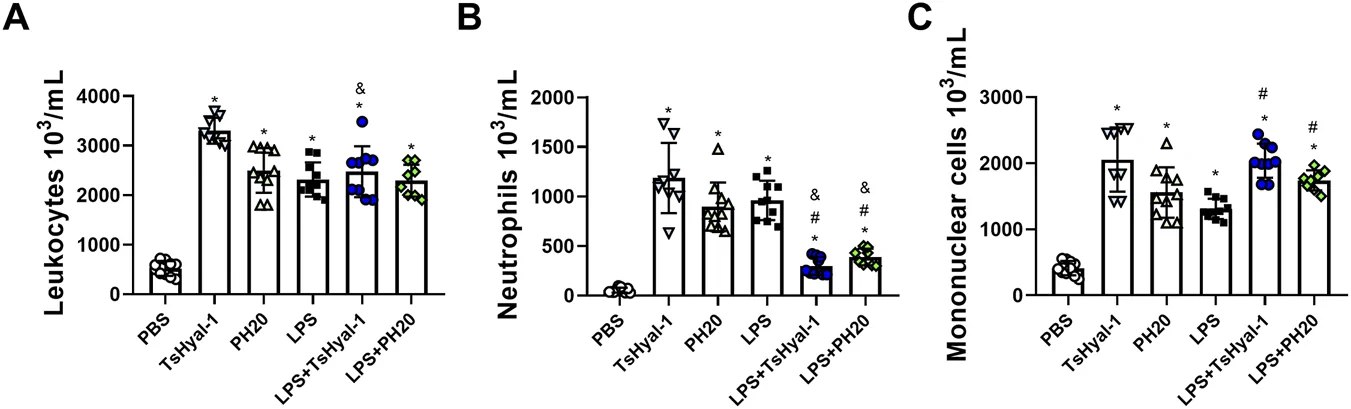
Hyaluronidase differentially modulates leukocyte recruitment during LPS-induced peritoneal inflammation. Mice were treated as follows: an initial intraperitoneal (i.p.) injection of PBS, TsHyal-1 or PH20 (11 TRU/mouse in 300 μL), or LPS (100 μg/kg in 300 μL), followed 15 min later by a second i.p. injection. Control, hyaluronidase-alone (TsHyal-1 or PH20), and LPS-alone groups received PBS as the second injection, whereas LPS-challenged mice received TsHyal-1 or PH20 (11 TRU/mouse). Peritoneal lavage fluids were collected 60 min after the second injection for total **(A)** and differential leukocyte counts, including neutrophils **(B)**, and mononuclear cells **(C)** Data are presented as mean ± standard deviation (SD) from two independent experiments (*n* = 5 mice per group per experiment; total *n* = 10 mice per group). Statistical analyses were performed using one-way ANOVA followed by Dunnett’s multiple-comparisons test. Predefined comparisons were performed using PBS, LPS, TsHyal-1, or PH20 as the corresponding reference groups, according to the biological question addressed. Differences were considered statistically significant at *p* < 0.05. Symbols indicate statistically significant differences relative to the corresponding reference group, with *p*-values equal to or lower than the largest value represented by each symbol: *p ≤ 0.0389 vs. PBS group; ^#^ *p* ≤ 0.0108 vs. LPS group; ^&^ *p* ≤ 0.0001 vs. the corresponding hyaluronidase-alone group (TsHyal-1 or PH20).

Peritoneal lavage was analyzed to assess leukocyte recruitment after mice received intraperitoneal LPS as an inflammatory stimulus, followed 15 min later by TsHyal-1 or PH20. Control groups received PBS, LPS alone, or TsHyal-1 or PH20 alone. As shown in Figure 1, TsHyal-1 and PH20 alone induced leukocyte recruitment (Figure 1A), mainly neutrophils (Figure 1B). Both hyaluronidases significantly increased total leukocyte recruitment compared with the PBS group (*p* < 0.0001 for both comparisons). LPS administration significantly increased total leukocyte counts relative to PBS control. In contrast, both hyaluronidases significantly reduced neutrophil accumulation in LPS-treated mice (both LPS + TsHyal-1 and LPS + PH20 vs. LPS, *p* < 0.0001) (Figure 1B). Compared with the PBS group, all treatments significantly increased mononuclear cell recruitment. Furthermore, animals receiving LPS in combination with TsHyal-1 or PH20 exhibited higher mononuclear cell recruitment than those treated with LPS alone (Figure 1C).

### 3.4 Hyaluronidase amplifies IL-6 and IL-1β production

**FIGURE 2:**
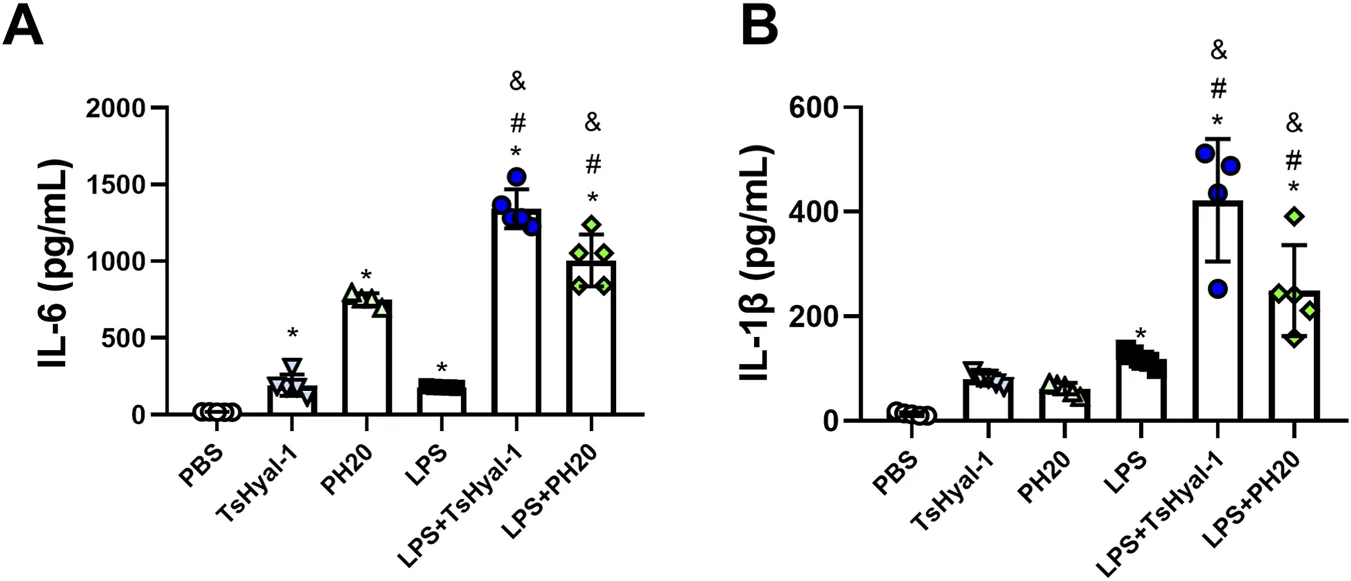
Hyaluronidase amplifies cytokine production during LPS-induced peritoneal inflammation. Mice were treated as described in Figure 1. Peritoneal lavage fluids were collected 60 min after the second i.p. injection, and IL-6 **(A)** and IL-1β **(B)** concentrations were quantified by ELISA. Data are expressed as mean ± SD (*n* = 5 mice per group), using biological samples obtained from two independent experiments according to the predefined experimental design. Statistical analyses were performed using one-way ANOVA followed by Dunnett’s multiple-comparisons test. Predefined comparisons were performed using PBS, LPS, TsHyal-1, or PH20 as the corresponding reference groups, according to the biological question addressed. Differences were considered statistically significant at *p* < 0.05. Symbols denote significant differences relative to the corresponding reference group, with *p*-values equal to or lower than the largest value represented by each symbol: *p ≤ 0.0486 vs. PBS group; ^#^ *p* ≤ 0.0055 vs. LPS group; ^&^ *p* ≤ 0.0023 vs. the corresponding hyaluronidase-alone group (TsHyal-1 or PH20).

LPS administration induced a robust inflammatory response in the peritoneal cavity, characterized by increased IL-6 and IL-1β levels compared with PBS controls (Figures 2A,B). In the absence of LPS, both TsHyal-1 and PH20 promoted increased IL-6 production, whereas only TsHyal-1 significantly increased IL-1β levels relative to the PBS group. When combined with LPS, both TsHyal-1 and PH20 further increased IL-6 production compared with LPS alone (both LPS + TsHyal-1 and LPS + PH20 vs. LPS, *p* < 0.0001). A similar effect was observed for IL-1β (LPS + TsHyal-1 vs. LPS, *p* < 0.0001; LPS + PH20 vs. LPS, *p* = 0.0055). Importantly, this cytokine amplification occurred concomitantly with reduced neutrophil recruitment (Figure 1), indicating that increased IL-6 and IL-1β levels occurred despite reduced neutrophil accumulation under the experimental conditions evaluated.

### 3.5 Histopathological features of inflammation following hyaluronidase treatment

**FIGURE 3:**
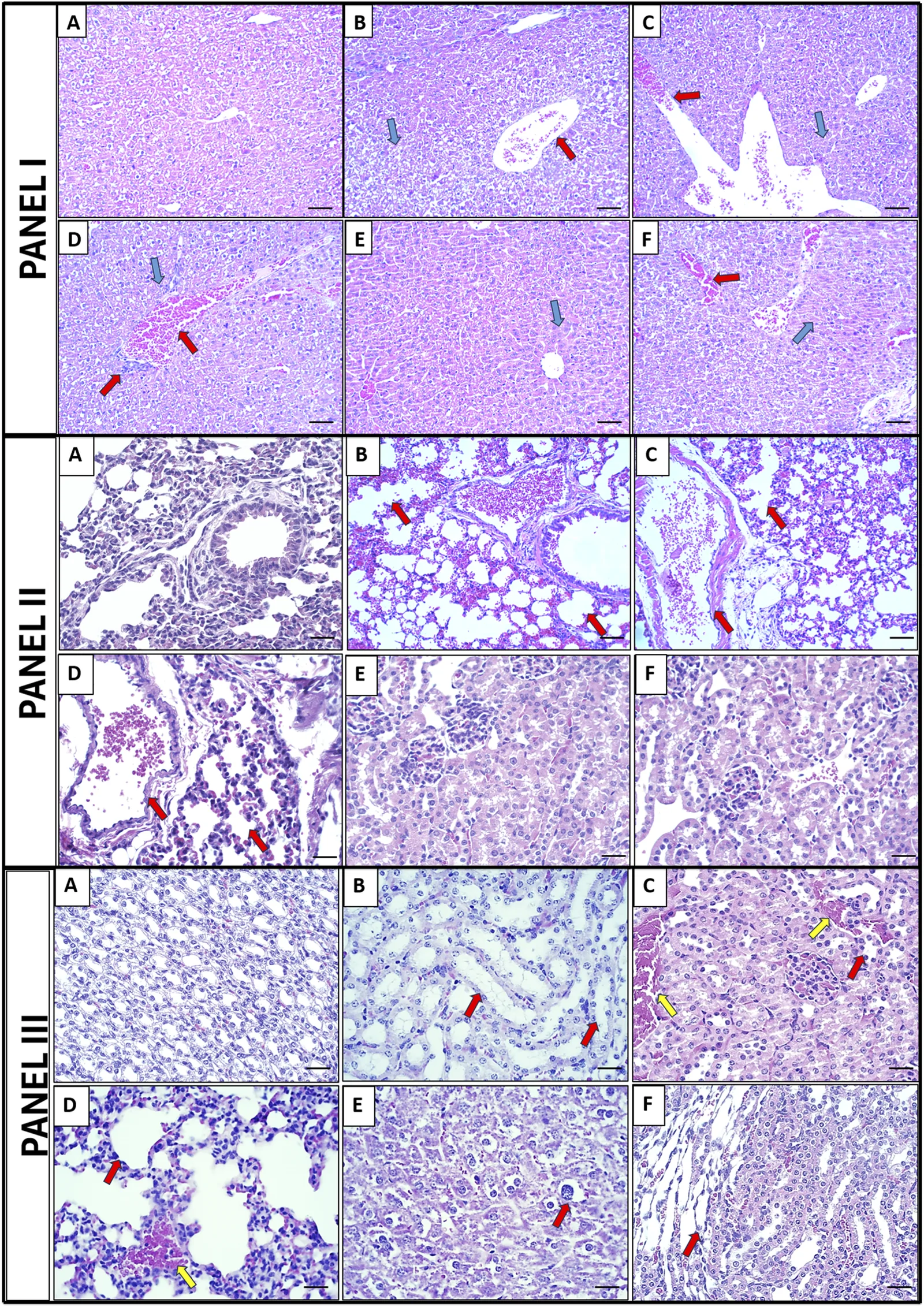
Histopathological analysis of liver, lung, and kidney following hyaluronidase treatment during LPS-induced peritoneal inflammation. Representative hematoxylin and eosin (H&E)-stained sections of liver **(I)**, lung **(II)**, and kidney **(III)** collected 60 min after the second i.p. injection from mice treated with PBS **(A)**, TsHyal-1 **(B)**, PH20 **(C)**, LPS **(D)**, LPS + TsHyal-1 **(E)**, and LPS + PH20 **(F)** Colored arrows indicate the main histopathological findings: **(I)** (liver): red arrows, sinusoidal congestion; blue arrows, disruption of hepatic architecture. **(II)** (lung): red arrows, interstitial thickening. **(III)** (kidney): red arrows, tubular alterations; yellow arrows, glomerular congestion. Images were acquired using a ×40 objective under the same imaging parameters (Scale bar = 20 μm). Histological evaluation was performed qualitatively by two independent blinded observers using tissues from all animals included in the study (n = 10 mice per group; total n = 60). Representative images illustrating the predominant qualitative histopathological findings.

Histopathological evaluation (Figure 3) was performed in liver (Figure 3I), lung (Figure 3II), and kidney (Figure 3III) tissues to assess tissue alterations associated with inflammation and their modulation by hyaluronidase.

Compared with PBS, all inflammatory stimuli induced tissue alterations of variable intensity (Figure 3). In the liver (Figure 3I), the LPS, TsHyal-1, and PH20 groups showed sinusoidal congestion and disruption of the hepatic architecture. In the LPS + hyaluronidase groups, these alterations appeared less pronounced, with qualitative differences consistent with partial preservation of the hepatic architecture, although residual sinusoidal congestion remained evident in the LPS + PH20 group (Figures 3I, F).

In the lung (Figures 3II, F), the LPS, TsHyal-1, and PH20 groups exhibited marked interstitial thickening and disruption of the alveolar architecture relative to the PBS group. In the LPS + hyaluronidase groups, these alterations were attenuated, with partial preservation of the pulmonary architecture, although qualitative differences relative to the LPS group remained subtle (Figures 3II, E,F).

In the kidney (Figures 3III, F), tubular alterations were observed across groups. Animals treated with LPS + hyaluronidase showed a tendency toward preservation of tubular architecture and less prominent glomerular congestion, particularly in the TsHyal-1 group (Figures 3III, E).

Complementary morphometric analysis did not reveal significant differences among experimental groups, which may reflect the intrinsic variability of this experimental model and the limited sensitivity of the assessed parameters. Overall, although qualitative examination suggested subtle differences among groups, these observations should be interpreted cautiously because complementary morphometric analyses did not show statistically significant differences.

### 3.6 Global protein profile remains stable despite inflammatory and enzymatic modulation

To assess potential proteolytic changes, peritoneal lavage samples were analyzed under native and denaturing electrophoretic conditions, including Tris-Tricine SDS-PAGE (Supplementary Figure S1).

Protein banding patterns in native PAGE (pH 8.6) (Supplementary Figure S1A), conventional SDS-PAGE (10%) (Supplementary Figure S1B), and Tris-Tricine SDS-PAGE (Supplementary Figure S1C) were comparable across all groups, with no consistent differences in band number, molecular weight distribution, or relative band intensity. A prominent band (∼60–70 kDa), likely corresponding to albumin, was identified in all samples.

No enrichment of low-molecular-weight protein fragments or differential banding patterns was detected across experimental groups. These findings are consistent with preservation of the global protein profile and do not support widespread proteolysis under the experimental conditions.

### 3.7 Low hyaluronan abundance and absence of detectable fragmentation in peritoneal lavage

Hyaluronan (HA) was detected at consistently low levels in peritoneal lavage across all experimental groups, with most measurements near or below the limit of quantification (LOQ = 0.284 µg). Absolute values ranged from approximately 0.07–0.61 µg, suggesting restricted HA availability within the peritoneal compartment under these conditions.

Electrophoretic analysis showed predominantly high-molecular-weight HA, with no detectable shift toward lower molecular weight species within the detection limits of the method (Supplementary Figures S2, S3). Bulk peritoneal lavage analysis did not detect HA fragmentation under the experimental conditions tested.

## 4 Discussion

This study demonstrates that scorpion venom hyaluronidase treatment was associated with condition-dependent changes in inflammatory parameters, unveiling an association between increased IL-6/IL-1β levels and reduced neutrophil accumulation under the experimental conditions evaluated. Under non-LPS conditions, enzymatic administration promoted neutrophil influx and modulated cytokine production, whereas in LPS-induced peritoneal inflammation, hyaluronidase further increased IL-6 and IL-1β levels while concomitantly reducing neutrophil accumulation. Hyaluronidase treatment was also associated with qualitative histopathological differences that should be interpreted cautiously because complementary morphometric analyses were not statistically significant. These findings suggest that inflammatory outcomes may reflect additional factors beyond cytokine levels; however, these factors were not directly investigated in the present study.

Hyaluronan (HA) levels in peritoneal lavage samples were consistently low across experimental groups and were frequently near or below the limit of quantification (LOQ), and electrophoretic analysis did not reveal detectable fragmentation or shifts in molecular weight distribution. Accordingly, bulk peritoneal lavage analysis did not detect HA fragmentation under the experimental conditions tested. The mechanistic interpretations proposed here should therefore be viewed as hypotheses generated from the experimental observations described herein rather than as direct evidence of the underlying mechanisms. Dedicated experimental studies using complementary approaches to evaluate extracellular matrix physical properties and dynamics, including biophysical analyses, will be important to better characterize the inflammatory phenotype associated with hyaluronidase treatment.

Although fragmentation of high-molecular-weight hyaluronan (HMW-HA) into low-molecular-weight species (LMW-HA) has been proposed as a mechanism linking hyaluronidase activity to pro-inflammatory signaling via TLR2 and TLR4 activation (Campo et al., 2012; Li et al., 2010; Silva de França and Tambourgi, 2023) (You et al., 2021), the present data neither support nor exclude localized or transient HA-dependent signaling. Tissue-localized HA staining or more sensitive analytical approaches will be required to formally evaluate this possibility.

The absence of detectable HA fragmentation also indicates that the biological responses observed cannot be readily attributed to detectable HA degradation under the experimental conditions evaluated. Studies on other venom enzymes, such as catalytically inactive phospholipases A_2_, have demonstrated that non-catalytic domains can exert biological activities through protein-protein interactions (de Oliveira et al., 2024). By analogy, hyaluronidases may exert immunomodulatory effects through pathways that are not exclusively dependent on HA degradation. Whether these involve non-catalytic interactions with cell-surface components or other pathways remains unresolved and will require dedicated experimental evaluation using recombinant enzymes with abolished catalytic activity or pharmacological inhibition. The associations identified in this study should therefore be interpreted as correlative rather than mechanistic, since the present experiments were not designed to establish causal pathways. Because hyaluronidases are enzymes, dose normalization was based primarily on catalytic activity (TRU), which was considered the most appropriate parameter for comparing enzymatic preparations. Nevertheless, since protein structure itself may contribute to biological effects independently of catalysis, the administered doses were also shown to be nearly equimolar (Supplementary Table S1). Therefore, the differences observed between TsHyal-1 and bovine PH20 are unlikely to be explained simply by disparities in protein amount and more likely reflect intrinsic biochemical and structural differences between the enzymes, although additional experimental studies using catalytically inactive variants will be necessary to distinguish catalytic from non-catalytic mechanisms. Residual endotoxin contamination in the enzyme preparations was not directly quantified and therefore cannot be formally excluded. Future investigations incorporating endotoxin quantification and catalytically inactive enzyme controls will be important to further define the mechanisms responsible for these findings. Although both male and female mice were included in this study, the experimental design and sample size were not intended to evaluate sex-dependent differences. Therefore, the present findings should be interpreted as representing the overall biological response rather than sex-specific effects.

Condition-dependent biological effects have also been described for other venom proteins (Clissa et al., 2024). For example, the snake venom disintegrin Alternagin-C promotes angiogenesis at low concentrations but inhibits it at higher concentrations (Clissa et al., 2024; dos Santos et al., 2020). Although mechanistically distinct from hyaluronidases, these observations reinforce the broader concept that venom proteins may exert biological activities beyond their canonical functions, consistent with the differential biological effects observed in the present study.

Hyaluronidases have been reported to modulate the extracellular matrix physical properties, including matrix viscosity and stiffness, water retention, osmotic pressure, and interstitial fluid pressure, thereby increasing tissue permeability and porosity (Weber et al., 2019; Ding et al., 2022; Chen et al., 2020). These changes have been reported to influence neutrophil recruitment and the local distribution of inflammatory mediators independently of HA fragment generation and could contribute to the identified association between increased IL-6/IL-1β levels and reduced neutrophil accumulation, although this possibility was not directly investigated in the present study.

Qualitative histopathological evaluation suggested subtle differences among groups; however, these findings should be interpreted cautiously because complementary morphometric analyses did not show statistically significant differences. The sinusoidal congestion observed in the liver is consistent with previous reports identifying this vascular alteration as a common feature of acute hepatic injury and inflammatory responses (Marzano et al., 2015). Although IL-6 and IL-1β levels were markedly elevated in LPS-challenged animals treated with hyaluronidase, neutrophil accumulation was significantly reduced, suggesting the possibility that structural and local tissue factors may influence leukocyte recruitment, although this hypothesis was not directly examined in the present study.

While the present study focused on IL-6 and IL-1β as key inflammatory mediators, future studies examining neutrophil-related chemokines, including CXCL1/KC and CXCL2/MIP-2, together with adhesion molecules such as ICAM-1 and VCAM-1, may help clarify the mechanisms underlying the patterns of neutrophil recruitment identified here.

The absence of detectable changes in the global protein profile of peritoneal lavage samples is consistent with this interpretation. No evidence of widespread proteolysis or major alterations in protein composition was detected, not supporting extensive matrix degradation as the predominant explanation for the inflammatory profile observed. Notably, although total leukocyte counts were unchanged, neutrophil infiltration was selectively reduced, suggesting differential modulation of leukocyte subsets. This pattern is consistent with preferential effects on neutrophil recruitment and/or migration, consistent with previous experimental observations demonstrating that changes in extracellular matrix biology can reduce leukocyte adhesion and tissue damage in localized inflammatory models (Fronza et al., 2014; Fronza et al., 2016).

The *in silico* analyses further support the notion that TsHyal-1 and bovine PH20 share a conserved catalytic framework despite substantial sequence divergence. Pairwise sequence alignment revealed relatively low overall sequence identity (34.3%) while demonstrating conservation of the catalytic core, indicating preservation of enzymatic function during evolutionary divergence. In contrast, the proteins differed in several sequence-derived structural features, including the distribution of cysteine residues, predicted N-linked glycosylation sites, and peripheral loop regions (Supplementary Figure S4). Although both enzymes contain twelve cysteine residues, their distinct distribution suggests differences in disulfide-bond organization and overall tertiary architecture. NetNGlyc analysis predicted six potential N-linked glycosylation sites for bovine PH20 and four for TsHyal-1, indicating differences in both the number and distribution of putative glycosylation sites. Because N-linked glycosylation has been reported to influence protein folding, stability, secretion, and substrate interactions, these differences could contribute to distinct biochemical properties. However, these predictions are based solely on primary sequence analysis and do not demonstrate glycan occupancy or functional significance *in vivo*.

These observations are consistent with the ProtParam analysis, which predicted only modest differences in physicochemical properties between TsHyal-1 and PH20. Thus, despite sharing a conserved catalytic framework, the two enzymes differ in several sequence-derived structural features while exhibiting only minor differences in their overall physicochemical characteristics. Whether these features contribute to their distinct biological profiles remains to be clarified. Accordingly, these computational predictions should be interpreted cautiously, as they do not establish a causal relationship with the experimental findings. Functional differences may also reflect post-translational modifications, experimentally unverified glycosylation patterns, or differences in substrate specificity that cannot be inferred solely from primary sequence analysis. Consequently, the distinct biological profiles of TsHyal-1 and PH20 should not be attributed solely to these sequence-based predictions.

Beyond its established clinical applications as a drug-dispersion enhancer (Bordon et al., 2015), hyaluronidase may also exert environment-dependent immunomodulatory effects, although their potential clinical implications remain unresolved.

The native *T. serrulatus* enzyme exhibited more pronounced immunomodulatory activity than the commercial bovine PH20, despite both enzymes being administered at equivalent catalytic activity (11 TRU) and nearly equimolar concentrations (0.9 µM, Supplementary Table S1). These observations suggest that structural characteristics may influence biological function beyond catalytic activity. Future dose-response investigations will be important to determine whether these differences persist at lower doses and under physiologically relevant *in vivo* exposures. In addition, studies employing catalytically inactive variants will be necessary to distinguish enzymatic from structure-dependent functional effects.

Traditionally characterized as a “spreading factor” that facilitates venom dissemination (Pessini et al., 2001), *T. serrulatus* venom hyaluronidase has been studied primarily in the context of envenomation. Inhibition of this enzyme prolongs survival and reduces lethality in experimental envenomation models (Horta et al., 2014; de Oliveira-Mendes et al., 2019), supporting its contribution to venom spread. However, accumulating evidence indicates a broader biological repertoire. Hyaluronidase from *Olivierus martensii* (previously *Buthus martensii*) modulates tumor cell adhesion and migration through matrix-CD44 interactions (Feng et al., 2008), while intranasal administration of bovine testicular and *T. serrulatus* hyaluronidases attenuates pulmonary fibrosis *in vivo* (Bitencourt et al., 2011). The present results further expand the current understanding of venom hyaluronidases by demonstrating differential changes in inflammatory parameters under the experimental conditions evaluated.

A conceptual parallel can be drawn from clinical observations in *Bothrops* envenomation, where restoration of matrix metalloproteinase/tissue inhibitor of metalloproteinase (MMP/TIMP) homeostasis following antivenom therapy has been associated with improved clinical outcomes (Ferreira Neves et al., 2026). Although derived from a distinct biological setting, these findings reinforce the concept that coordinated regulation of extracellular matrix dynamics, rather than the activity of a single mediator, may influence inflammatory outcomes.

Future investigations incorporating blinded semiquantitative histological scoring, quantitative morphometry, and immunostaining for specific leukocyte populations, such as neutrophils (e.g., anti-Ly6G) and macrophages (e.g., anti-F4/80), will further refine the histopathological characterization of these responses.

Taken together, these findings indicate that hyaluronidase treatment was associated with context-dependent changes in inflammatory parameters. The observed association between increased IL-6/IL-1β levels and reduced neutrophil accumulation, together with the absence of detectable HA fragmentation in bulk peritoneal lavage, is compatible with the possibility that hyaluronidase treatment may influence inflammatory responses through mechanisms that were not directly evaluated in the present study and may not be fully explained by detectable HA fragmentation in bulk peritoneal lavage samples, although the underlying molecular pathways remain to be clarified.

## 5 Conclusion and perspectives

This study shows that the effects of native scorpion venom hyaluronidase on inflammatory parameters differ between non-LPS conditions and LPS-induced peritoneal inflammation. Under non-LPS conditions, hyaluronidase promoted neutrophil recruitment and increased IL-6 and IL-1β levels, accompanied by moderate tissue alterations. In contrast, during LPS-induced peritoneal inflammation, treatment was associated with a non-canonical inflammatory phenotype characterized by further cytokine amplification alongside reduced neutrophil accumulation, whereas qualitative histological observations suggested subtle differences among groups that were not confirmed by complementary morphometric analysis. The observed association between increased IL-6/IL-1β levels and reduced neutrophil accumulation may reflect additional factors beyond soluble cytokine levels, although these factors were not directly investigated. The data are compatible with the possibility that hyaluronidase treatment may influence inflammatory responses through alterations in the extracellular environment, although the underlying mechanism remains to be experimentally investigated.

These findings represent an initial step toward understanding the immunomodulatory potential of scorpion venom hyaluronidase. The observations reported here provide a foundation for future investigations, including studies with catalytically inactive enzymes, detailed analyses of chemokines and adhesion molecules, and direct assessment of extracellular matrix physical properties. The observed association between increased IL-6/IL-1β levels and reduced neutrophil accumulation merits dedicated mechanistic evaluation and may contribute to our understanding of inflammatory regulation in complex biological systems.

## Data Availability

The original contributions presented in the study are included in the article/Supplementary Material, further inquiries can be directed to the corresponding author.
