# Supplementary material for: Hyaluronidase treatment is associated with increased cytokine levels and reduced peritoneal neutrophil accumulation during LPS-induced inflammation
Source: Front Pharmacol. 2026 Aug 21;17:1869630. doi: 10.3389/fphar.2026.1869630 (PMC13541562; doi:10.3389/fphar.2026.1869630)
Supplement: Supplementary file 1 [file Supplementaryfile1.docx]

Supplementary Material

Supplementary Figures

**Supplementary Figure S1.** **Electrophoretic analysis of peritoneal lavage proteins under native and denaturing conditions. (A)** Native PAGE (12%, pH 8.6). Proteins were separated under non-denaturing conditions using Tris-glycine running buffer (pH 8.6). Electrophoresis was performed at 130 V (107–72 mA) for 1h 15 min. **(B)** SDS-PAGE (10%). Proteins were separated under standard denaturing conditions. Electrophoresis was performed at 60 V (42–37 mA) for 30 min, followed by 100 V (85–15 mA) for 1 h 45 min. **(A-B)** Gels were stained with 0.02% Coomassie Blue G-250. **(C)** Tris-Tricine SDS-PAGE (10%) with silver staining. Proteins were resolved under denaturing conditions optimized for low-molecular-weight detection. Silver staining was used for high-sensitivity detection. Legend: TI, soybean trypsin inhibitor (~20 kDa; ~pI 4.5; 0.5 µg); A, albumin (~66 kDa; ~pI 4.7; 0.5 µg); Cs, casein (a group of phosphoproteins; ~pI 4.4–5.2; 0.5 µg); Group legend (5 µg/lane): P, PBS; N, native hyaluronidase TsHyal-1 from *Tityus serrulatus* scorpion venom; C, bovine PH20; L, LPS; LN, LPS + N; LC, LPS + C; #, biological replicate. Molecular mass standards (kDa): Sigma M3546 (1.06–26.6 kDa) and Bio-Rad #1610374 (10–250 kDa).

**Supplementary Figure S2.** **Colorimetric quantification of hyaluronan (HA) in peritoneal lavage samples. Upper panel:** Representative Stains-all microplate showing HA standards (0.1–24.0 µg) and experimental samples, with concentration-dependent color shift from pink to blue. Sample nomenclature reflects biological origin and processing stage: L1, L2 and P1–P5, individual biological replicates (“n samples”) yielding detectable glycosaminoglycan (GAG) pellets after the first precipitation; LNx and Px (“x” samples), pooled samples from five animals processed in parallel; unnumbered samples, pellets obtained after reprocessing pooled supernatants; (*) fractions, pellets derived from additional centrifugation of secondary supernatants, representing highly soluble GAG fractions. **Lower panel:** HA standard curve (0–2.0 µg; y = 0.128x + 0.1191; R² = 0.9983), with limit of detection (LOD) = 0.094 µg and limit of quantification (LOQ) = 0.284 µg, and corresponding sample quantification.

**Supplementary Figure S3.** **Electrophoretic analysis of hyaluronan (HA) under non-denaturing conditions.** **(A)** PAGE (15%) of HA-enriched fractions from peritoneal lavage fluids stained with Stains-all. Samples were resolved in TBE buffer (pH 8.3) using a stepped voltage protocol (70 V for 30 min followed by 130 V for 1 h). Lanes: P, PBS; N, native hyaluronidase TsHyal-1 from *Tityus serrulatus* venom; C, bovine PH20; L, LPS; LN, LPS + N; LC, LPS + C; HAh2 and HAh4, HA (2 µg) incubated with PH20 (2 or 4 TRU, respectively) at 37 °C for 15 min; HA, untreated hyaluronan (2 µg). **(B)** Control gel run under identical conditions with purified glycosaminoglycan standards to validate glycosaminoglycan migration patterns and staining specificity. Hyaluronan (HA), chondroitin-4-sulfate (C4S), dermatan sulfate (DS), and chondroitin-6-sulfate (C6S) were applied at increasing concentrations (µg/lane, as indicated).

**Supplementary Figure S4. Pairwise sequence alignment of bovine PH20 (top; UniProt ID: Q7YS45) and TsHyal-1 from *Tityus serrulatus* (bottom; UniProt ID: P85841).** Conserved residues are shown as white letters on a black background, whereas non-conserved residues are shown on a white background. Conserved cysteine residues are highlighted in blue boxes and numbered according to their order in TsHyal-1 or bovine PH20. Magenta diamonds below the blue boxes indicate cysteine residues unique to TsHyal-1, whereas green triangles indicate cysteine residues unique to bovine PH20. Red squares identify conserved residues involved in positioning the carbonyl group of the substrate acetamido moiety required for catalysis. The catalytic Glu residue is indicated by a red star below the corresponding position in the alignment. The Tyr residue associated with hyaluronan substrate specificity is highlighted by a yellow square and indicated by a yellow star below the alignment. Predicted N-linked glycosylation sites (Asn-X-Ser/Thr) identified using the NetNGlyc 1.0 Server are highlighted in magenta boxes in TsHyal-1 and green boxes in bovine PH20. Pairwise sequence alignment was performed using the MultAlin Interface Page; sequence identity and similarity were calculated using the LALIGN algorithm, and the alignment was rendered using the ESPript 3.0 server.

Supplementary Methods

## S1.1 Hyaluronan Analysis in Peritoneal Lavage Fluid

Hyaluronan (HA) present in peritoneal lavage samples was purified, quantified, and characterized following selective enrichment of glycosaminoglycans. To improve HA recovery from complex biological matrices, we developed a proteolysis-guided enrichment approach that integrates enzyme-specific digestion parameters with controlled protein precipitation. This strategy combines enzymatic digestion with selective removal of protein components to promote dissociation of protein-hyaluronan complexes and enhance recovery of HA in the soluble fraction, while minimizing protein-associated interference and preserving polymer integrity.

### S1.1.1 Protein Precipitation and Hyaluronan Enrichment

Peritoneal lavage samples (500 µL in PBS; ~350 µg total protein) from the selected subset of animals (n = 5 per experimental group) were initially processed as individual biological replicates (hereafter denoted as “n samples”). Because hyaluronan recovery was consistently low, equal aliquots from animals within each experimental group were additionally pooled ("x samples") and processed in parallel to increase HA recovery. To facilitate interpretation of the electrophoretic profiles, the nomenclature adopted for each recovered fraction is described below.

Samples were subjected to proteolytic digestion with papain (1 µg/µL; enzyme-to-protein ratio 1:20), previously activated in PBS (pH 7.2) containing 2 mM EDTA and 5 mM cysteine, to release glycosaminoglycans (GAGs) from protein complexes. Incubation was performed at 37 °C for 15 min followed by 55 °C for 15 h, after which NaCl concentration was adjusted to 0.3 M.

Proteins were precipitated by addition of trichloroacetic acid (TCA) to a final concentration of 10%, followed by incubation at 4-8 °C for 30 min and centrifugation at 10,000 *× g*, 4 °C for 15 min. The resulting supernatant was neutralized with 2 M Tris-HCl (pH 8.8), and GAGs were precipitated by addition of three volumes of cold ethanol, readjusted to 0.3 M NaCl, incubated at −20 °C for 20 h, and centrifuged under the same conditions, yielding GAG-enriched pellets derived from either individual samples (“n”) or pooled samples (“x”).

For clarity, GAG fractions were classified according to their origin and processing stage. Primary pellets (“n” or “x”) correspond to fractions obtained after the first ethanol precipitation from individual or pooled samples, respectively. Secondary pellets (unnumbered) were obtained after reprocessing the combined supernatants generated during the initial precipitation steps, representing GAG fractions not recovered during the first precipitation. Highly soluble fractions (*) correspond to additional pellets recovered after further high-speed centrifugation of secondary supernatants.

To maximize HA recovery, supernatants remaining after the initial precipitation were combined within each experimental group, readjusted to 0.3 M NaCl, incubated at −80 °C for 64 h, and centrifuged (10,000 × g, 4 °C, 30 min), yielding secondary pellets (unnumbered samples). In the PBS group, an additional pellet was obtained after further centrifugation (14,000 × g, 4 °C, 10 min), designated as sample P*.

All resulting pellets were resuspended in 500 µL of ultrapure water, concentrated to 50 µL, and desalted by five cycles of dilution and concentration using Amicon Ultra centrifugal filters (3 kDa MWCO). The retained fractions (>3 kDa), enriched in HA, were dried in a SpeedVac concentrator and resuspended in 10 µL of ultrapure water for downstream analyses.

### S1.1.2 Spectrophotometric Quantification of Hyaluronan

Hyaluronan (HA) was quantified using the cationic dye Stains-all (E9379, Sigma-Aldrich, St. Louis, MO, USA), based on the spectral shift that occurs upon binding to non-sulfated glycosaminoglycans, as described by Fagnola et al. (2009). The dye solution (0.01%) was prepared by dissolving 1 mg in 8.6 mL of ultrapure water and 1.4 mL of methanol. Reactions were performed in non-treated, flat-bottom 96-well plates (final volume 200 µL), containing 1 µL of sample, 139 µL of ultrapure water, and 60 µL of dye solution (final concentration: 0.03 mg/mL). Plates were incubated for 30 min at room temperature, protected from light, and absorbance was measured at 640 nm. Blanks consisted of dye and ultrapure water only. Samples analyzed corresponded to the GAG-enriched fractions derived from primary pellets (samples “n” and “x”), secondary pellets (unnumbered samples), and highly soluble fractions (P*), as described in Section 2.8.1.

Calibration curves were prepared using hyaluronan from umbilical cord (H1876, Sigma-Aldrich) in two ranges (0.1–2 µg/mL and 1–24 µg/mL), matching assay conditions. Absorbance values showed high linearity (R² ≥ 0.99). The limit of detection (LOD) and limit of quantification (LOQ) were determined from the standard curve, and HA concentrations were calculated by interpolation within the linear range. Results were expressed as total hyaluronan content per experimental group.

### S1.1.3 Electrophoretic Analysis of Hyaluronan

The molecular profile of HA-enriched fractions was evaluated by PAGE under non-denaturing conditions, according to Ikegami-Kawai & Takahashi (2002). Samples (8 µL) were mixed with sucrose-containing loading buffer (5× TBE with 2 M sucrose; 4:1 (v/v) and applied (10 µL) to 15% acrylamide and 0.5% N,N'-methylene bisacrylamide gels prepared in 0.1 M Tris-borate buffer containing 1 mM Na₂EDTA (TBE, pH 8.3). Electrophoresis was performed in TBE using a stepped voltage protocol (70 V for 30 min followed by 130 V for 1 h; maximum 75 mA and 10 W per gel). Hyaluronan (2 µg per lane) was used as a reference standard. To generate controlled fragmentation patterns, HA incubated with bovine testicular hyaluronidase (2 or 4 TRU, 37 °C for 15 min) was applied on this gel. Bromophenol blue (0.005% in TBE containing 0.4 M sucrose) was used as a tracking dye in separate lanes. Glycosaminoglycans were detected by staining with 0.005% Stains-all in 50% ethanol under light-protected conditions for 1 h at room temperature, followed by destaining in 10% ethanol for 10 min. Gels were documented and migration profiles compared across groups.

A parallel control gel containing purified glycosaminoglycan standards, including hyaluronan (H1876 – hyaluronic acid sodium salt from human umbilical cord), chondroitin sulfate (C4S; CS-A; C6737 – chondroitin sulfate sodium salt from bovine cartilage; 57% C4S and 43% C6S), dermatan sulfate (DS; CS-B; C3788 – chondroitin sulfate B sodium salt from porcine intestinal mucosa), and chondroitin-6-sulfate (C6S; CS-C; C4384 – chondroitin 6-sulfate sodium salt from shark cartilage) (Sigma-Aldrich, St. Louis, MO, USA), applied at increasing concentrations (0.1-4.0 µg per lane), was used to validate migration behavior and staining specificity among different glycosaminoglycan species.

Supplementary Tables

**Supplementary Table S1.** **Calculation of activity-matched and nearly equimolar doses of *Tityus serrulatus* hyaluronidase (TsHyal-1) and bovine PH20 used in the *in vivo* experiments.**

| **Parameter** | **TsHyal-1**  **(*T. serrulatus*)** | **Bovine PH20** |
| --- | --- | --- |
| **Absorbance at 280 nm (A_280_)** | 0.456 | 0.220 |
| **Extinction coefficient (ε, mL·mg⁻¹·cm⁻¹)*** | 2.594 | 1.833 |
| **Estimated protein concentration (mg/mL)** | 0.176 | 0.120 |
| **Volume used for dose preparation (mL)** | 1.425 | 2.364 |
| **Protein mass recovered (µg)** | 250.5 | 283.7 |
| **Total TRU** | 228.1 | 226.1 |
| **Final resuspension volume (mL)** | 6.3 | 6.3 |
| **Final protein concentration (µg/mL)** | 39.8 | 45.0 |
| **Molecular weight (Da)*** | 44,547.14 | 49,669.70 |
| **Final molar concentration (µM)** | 0.892 | 0.907 |
| **Dose administered per animal (300 µL)** | 11.9 µg (0.268 nmol) | 13.5 µg (0.272 nmol) |
| **TRU administered per animal (300 µL)** | 10.9 | 10.8 |

*Protein molecular weights and extinction coefficients were predicted using ProtParam (ExPASy). All remaining values were calculated from experimental measurements obtained in this study.

**Supplementary Table S2.** **Comparative structural, physicochemical, and biochemical characteristics of *Tityus serrulatus* hyaluronidase (TsHyal-1) and bovine PH20.**

| **Parameter** | **TsHyal-1 (*T. serrulatus*)** | **Bovine PH20** |
| --- | --- | --- |
| **Primary sequence and structural properties** |  |  |
| UniProt accession† | P85841 | Q7YS45 |
| Sequence length (aa)† | 384 | 439 |
| Sequence identity (%)* | 34.3 | – |
| Sequence similarity (%)* | 67.7 | – |
| Predicted N-glycosylation sites (N-X-S/T)** | 4 | 6 |
| **Predicted physicochemical properties** |  |  |
| Theoretical pI§ | 8.75 | 8.34 |
| Instability index§ | 35.02 | 37.56 |
| Aliphatic index§ | 80.65 | 84.58 |
| GRAVY (grand average of hydropathicity)§ | −0.407 | −0.288 |
| Cysteine residues§ | 12 | 12 |
| Negatively charged residues (Asp + Glu)§ | 40 | 42 |
| Positively charged residues (Arg + Lys)§ | 48 | 46 |
| **Biochemical properties from literature** |  |  |
| Optimum temperature (°C)# | 40 | Not reported |
| Temperature range with high activity (°C)# | 30–50 | Not reported |
| Optimum pH# | 6.0 | 5.5–6.0 |
| Active pH range# | 4.0–8.5 | 4.0–7.0 |

**†** Primary sequences were retrieved from UniProt.

***** Sequence identity and similarity were determined from the alignment between TsHyal-1 and bovine PH20 using the LALIGN algorithm (<https://www.ebi.ac.uk/jdispatcher/psa/lalign>) (Madeira et al., 2024).

**§** Physicochemical parameters were predicted using the ProtParam tool available at the ExPASy server (<https://web.expasy.org/protparam/>).

**#** Biochemical properties of TsHyal-1 were obtained from Pessini et al. (2001). Reported pH optima for bovine testicular hyaluronidase vary depending on purification procedure, ionic strength, and assay conditions (Gorham et al., 1975).

** Predicted using the NetNGlyc 1.0 server (Gupta & Brunak, 2002).
